# A quaternary ammonium silane antimicrobial triggers bacterial membrane and biofilm destruction

**DOI:** 10.1038/s41598-020-67616-z

**Published:** 2020-07-03

**Authors:** Umer Daood, Jukka P. Matinlinna, Malikarjuna Rao Pichika, Kit-Kay Mak, Venkateshbabu Nagendrababu, Amr S. Fawzy

**Affiliations:** 1 Division of Clinical Dentistry, Faculty of Dentistry, School of Dentistry, International Medical University Kuala Lumpur, 126, Jalan Jalil Perkasa 19, Bukit Jalil, 57000 Wilayah Persekutuan Kuala Lumpur, Malaysia; 2 Dental Materials Science, Applied Oral Sciences and Community Dental Care, Faculty of Dentistry, The University of Hong Kong, 34 Hospital Road, Sai Ying Pun, Hong Kong, SAR People’s Republic of China; 3 Department of Pharmaceutical Chemistry, School of Pharmacy, International Medical University Kuala Lumpur, 126, Jalan Jalil Perkasa 19, Bukit Jalil, 57000 Wilayah Persekutuan Kuala Lumpur, Malaysia; 4 UWA Dental School, University of Western Australia, Nedlands, WA 6009 Australia

**Keywords:** Microbiology, Molecular biology, Health care, Materials science

## Abstract

To study the antimicrobial effects of quaternary ammonium silane (QAS) exposure on *Streptococcus mutans* and *Lactobacillus acidophilus* bacterial biofilms at different concentrations. *Streptococcus mutans* and *Lactobacillus acidophilus* biofilms were cultured on dentine disks, and incubated for bacterial adhesion for 3-days. Disks were treated with disinfectant (experimental QAS or control) and returned to culture for four days. Small-molecule drug discovery-suite was used to analyze QAS/Sortase-A active site. Cleavage of a synthetic fluorescent peptide substrate, was used to analyze inhibition of Sortase-A. Raman spectroscopy was performed and biofilms stained for confocal laser scanning microscopy (CLSM). Dentine disks that contained treated dual-species biofilms were examined using scanning electron microscopy (SEM). Analysis of DAPI within biofilms was performed using CLSM. Fatty acids in bacterial membranes were assessed with succinic-dehydrogenase assay along with time-kill assay. Sortase-A protein underwent conformational change due to QAS molecule during simulation, showing fluctuating alpha and beta strands. Spectroscopy revealed low carbohydrate intensities in 1% and 2% QAS. SEM images demonstrated absence of bacterial colonies after treatment. DAPI staining decreased with 1% QAS (*p* < 0.05). Fatty acid compositions of dual specie biofilm increased in both 1% and 2% QAS specimens (*p* < 0.05). Quaternary ammonium silane demonstrated to be a potent antibacterial cavity disinfectant and a plaque inhibitor and can be of potential significance in eliminating caries-forming bacteria.

## Introduction

Ninety-five percent of the bacteria present inside the oral cavity exist as plaque biofilms. The constant supply of nutrients derived from the oral cavity enables these biofilms to survive and adhere to tooth surfaces^1^. Genetic studies have revealed that biofilm formation occurs in multiple steps and involves quorum sensing signaling between bacterial cells driven by transcription of different set of genes in different bacterium^2^. The central part of the biofilm matrix represents an anaerobic environment with reduced oxygen, pH levels and nutrient availability. The biofilm spatial organisation, which consists of exopolysaccharides, is neutral or polyanionic due to the presence of uronic acids or ketal-linked pyruvates^3^. Caries development is a direct consequence of enrichment of acid tolerant bacterial colonies which propagate in dental biofilms in response to prolonged periods of low pH. The acid production from the acid tolerant bacteria cause demineralization of the enamel and dentine surface. Greater proportion of acidogenic *Lactobacilli* and *Streptococci* populations appear predominantly in a cariogenic biofilm circumventing the dissolution process of tooth substrate^4,5^. Previous studies reported that *Streptococcus sorbinus* and *Streptococcus mutans* have strong links with smooth surface caries^6–10^.

Most bonding procedures using dentine adhesives are performed on carious surface. This imperfect bonding to carious dentin produces inferior results irrespective of the adhesive system employed^11^. The low bond strength may be attributed to wetness, tubular occlusion, loss of minerals and degradation of the bonded interface by matrix metalloproteinases (MMP)^12,13^. Hence, new strategies are required to intercept caries^14^ with new antimicrobial agents^15,16^. Optimal cavity preparation requires maximum removal of infected carious tissues with minimal intervention and preservation of the tooth structures^17^. The conservative approach for caries removal mandates retention of this caries-affected dentin to avoid pulpal exposure^18^.

Quaternary ammonium silane (QAS; KHG FiteBac® Technology, Marietta, GA, USA) is an organic contact-killing agent that possesses broad spectrum antimicrobial activities with low cytotoxicity^19,20^. The quarternary ammonium amphiphiles have functional end-OH groups on their surfaces which can be surface modified (by use of acids) to activate the –OH groups^21^. The antimicrobial activity of quaternary ammonium silane is derived from its –C_18_H_37_ lipophilic alkyl chain which penetrates bacterial cell walls and membranes causing autolysis^22^. These compounds have shown their effectiveness in reduction of bacterial growth in a wide range of applications including textiles^23^, medical devices^24,25^, and most importantly dental materials^26^. The presence of an ammonium salt and both positive and negative charges in its molecular structure provide hydrophilic nature, i.e. absorbing polar solvents^26^. The lysis of bacterial cells by binding to the cell wall is an antibacterial mechanism of quaternary ammonium salt widely considered to be “contact killing”^27^ in presence of long lipophilic alkyl chain as mentioned previously. Apart from its antibacterial potential, QAS also demonstrates anti-MMP activity^15^ and optimum bond strength after its application to acid-etched dentin prior to adhesive bonding^19^. Surface adhesins derived from *Streptococcus mutans* are anchored on the bacterial cell surface via sortase A^28^, a transpeptidase that covalently links the LPXTGX motif-containing surface proteins to the cell wall of Gram-positive bacterial^29^. Sortase A plays a critical role in modulating the surface properties of bacteria that adhere to tooth surface and contributes to the carcinogenicity of *Streptococcus mutans*^30^.

The objective of the present laboratory study was to study the antimicrobial effects of QAS exposure on *Streptococcus mutans* and *Lactobacillus acidophilus* bacterial biofilms at different concentrations. The antibacterial analysis and QAS-induced membrane protein damage in bacteria were understood at molecular level using computational molecular simulations. The null hypotheses tested were that the QAS cavity disinfectant has (i) no antimicrobial effect on dual-species biofilms, and (ii) no adverse effect on the demineralized dentin collagen matrix.

## Results

**Figure 1:**
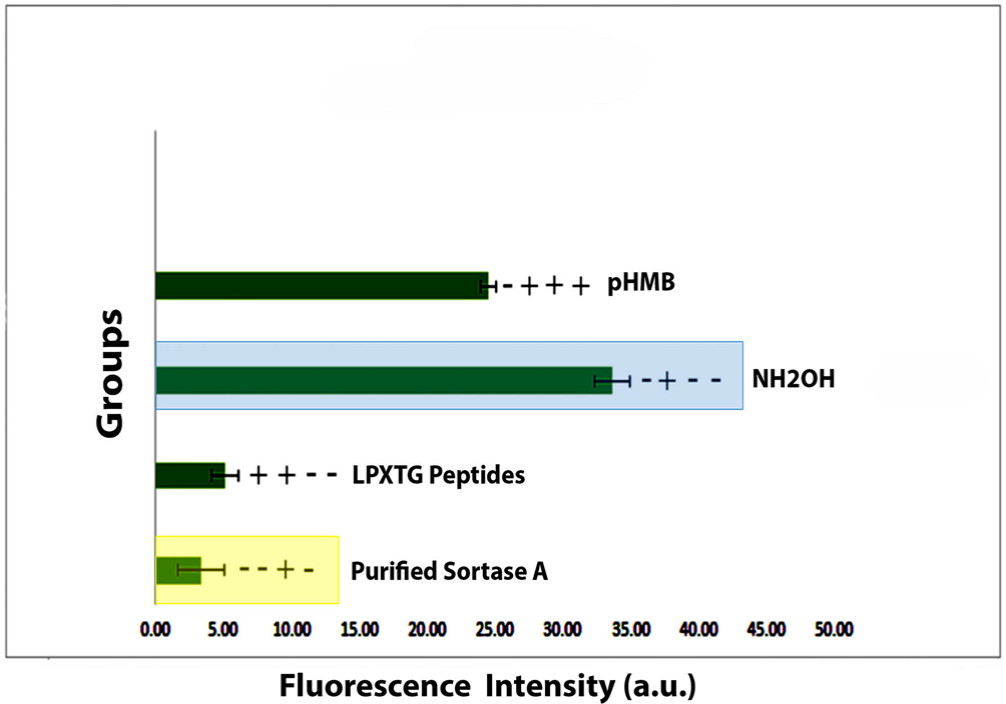
Purified sortase A analysis in vitro after incubation with the sorting substrate Dabcyl-QALPETGEE-Edans. Addition of 0.2 M NH_2_OH increased fluorescence intensity, whereas the addition of pHMB reduced the fluorescence intensity. Marked difference seen in purified sortase A fluorescence intensity (*p* < 0.05).

Fluorescence was increased sharply in 0.2 M NH_2_OH indicating an efficient LPXTG peptide indicating that purified sortase A possessed native LPXTG peptide cleavage activity. 2% QAS dilution significantly reduced and inhibited sortase A with an IC50 value of 3.3 ± 2.7 μM, a more potent value as compared to pHMB positive control, IC50 = 24.5 ± 4.1 μM (Fig. 1).

**Figure 2:**
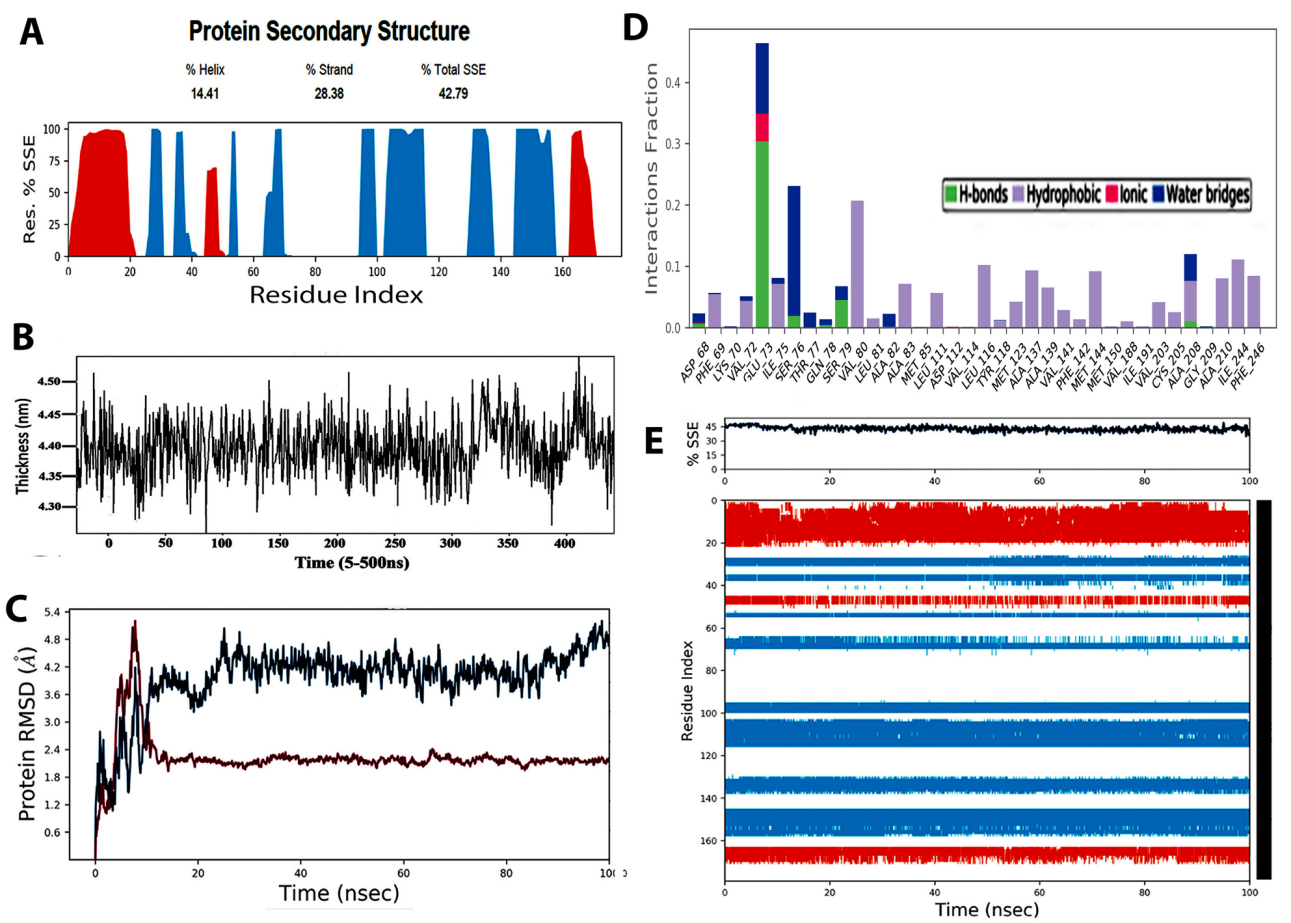
(**A**) Protein secondary structure elements (SSE) including alpha-helices and beta-strands were monitored throughout the simulation. The plot represents SSE distribution by residue index throughout the protein structure. All protein frames were first aligned on the reference frame backbone. Energy minimization was ensured to avoid inappropriate geometry and against steric clashes. Typically, it is observed that the tails (*N*- and *C*-terminal) fluctuate more than any other part of the protein. Secondary structure elements such as alpha helices and beta strands are usually more rigid than the unstructured part of the protein, and thus fluctuate less than the loop regions. (**B**) Restrictive simulation process of the lipid membrane with interaction energy and contact with the membrane. This was presented as the morphology of the upper lipid membrane layer with average z coordinate value of the surface set to 0. (**C**) The RMSD was calculated based on atom selection. Monitoring the RMSD of a protein provides insights into its structural conformation throughout the simulation. RMSD analysis indicates if the simulation has equilibrated; its fluctuations towards the end of the simulation are around thermal average structure. Since many molecules dock into the binding pocket, the spheres formed within 0.1–0.3 nm root mean square division established the crystal structure and maximum orientations. Changes in the order of 0.1–0.3 nm are acceptable for small, globular proteins. Changes larger than that value, however, indicate that the Sortase A protein undergoes a large conformational change during the simulation. Ligand RMSD (right Y-axis) indicates how stable the ligand is with respect to the protein and its binding pocket. (**D**) The stacked bar charts are normalized over the course of the trajectory: for example, a value of 0.7 suggests that 70% of the simulation time the specific interaction is maintained. Values over 1.0 are possible as some protein residue may make multiple contacts of same subtype with the ligand. The plot summarizes the SSE composition for each trajectory frame over the course of the simulation (**E**) The plot below summarizes the SSE composition for each trajectory frame over the course of the simulation.

The plot in Fig. 2 shows the root-mean-square deviation (RMSD) evolution of the Sortase A protein (left Y-axis). The reference frame for biofilm organization was used to align all protein frames and RSMD calculation was based on atom selection. Structural conformation insights for RSMD were monitored throughout the simulation. RMSD analysis indicates if the simulation has equilibrated.

**Figure 3:**
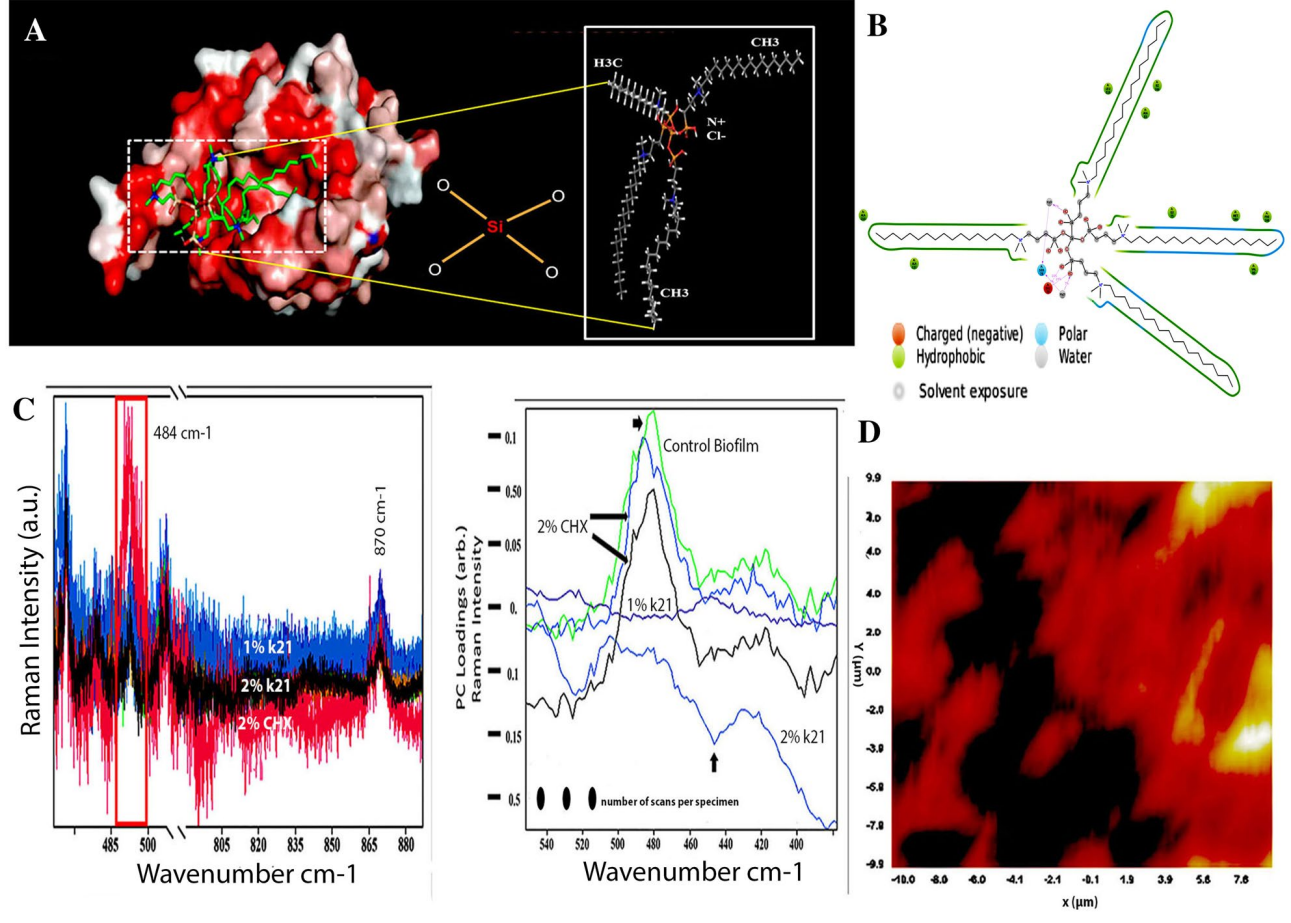
(**A**) Results of molecular docking simulation of QAS 1% on crystal structure of SrtA indicating a complex indicating a predicated interaction mode of QAS catalytic center of SrtA. The structure was generated from molecular coordinates from the Protein Data Bank, PDB ID. Subset proposed chemical formula of the QAS molecule. The docking shown in figure is typically performed on the basis of the known Sortase-A crystal structure and the SrtA-quaternary ammonium substrate complexes. The polar capabilities of QAS has enabled it to form charge-charge interactions that can insert with the binding pocket of SrT-A. (**B**) A schematic of detailed ligand atom interactions with the protein residues. Interactions that occur more than 5.0% of the simulation time in the selected trajectory (0.00 through 100.00 ns), are shown. (**C**) Raman spectra of dual specie biofilms grown on demineralized dentine specimens and treated with different concentrations of QAS and CHX disinfectants. Spectral differences of control and treated specimens can be seen in the 484 cm^−1^ region after normalization. Labelled bands present in the spectra are discussed in the text. Spectra are shifted to avoid overlap between the groups. The spectral lines are quantitative detection with each data point corresponding to the average signal collected from different groups. Raman spectra and the corresponding section corrected for orientation in a side-by-side image. For better comparability of the two measurements, different colours were chosen for the Raman spectrum of the dual specie bacterial biofilms. The molecules within the aromatic and functional groups have polarized electrons as a result of double bonds and free electrons which resulted in increased Raman shifts inside the specimens. The bands refer to the glycosidic link or ring breath of possible polysaccharides which typify the changes seen within the biofilm as a result of disinfectant treatment. These features are specific for polysaccharides (COC stretching and the anomeric C (1)-H deformations of α (1 → 4) glycosidic links) linked by 1–4 glycosidic bonds (amylose, amylopectin, glycogen). This finger print region attributed to bacterial carbohydrate via CO and CC stretching and bending vibrations showing similar changes as per our previous studies, this time also with 1% QAS molecules. (D) Raman image of intact dual specie cells with dark shading representing peak intensities at 484 cm^−1^ region corresponding loadings plot.

The fluctuations happening at the end of simulation indicate an average structure which is thermally stable. The crystal structure and formation of maximum orientation in spheres was formed within 0.1–0.3 nm root mean square division after docking of many molecules into the binding pocket. This change of order ranging between 0.1–0.3 nm is considered acceptable for small globular proteins with larger changes pointing a conformational change in Sortase A protein during simulation. The Y-axis RMSD ligand indicated the stability of the ligand with respect to the protein and its binding pocket. When the protein–ligand is first aligned on protein structure of the reference, the RMSD of heavy atoms is measured to construct the ‘Ligand-fit-Protein’ plot. It is most likely that the ligand has diffused away from its bonding site. As seen in the plot, the areas of protein are indicated as fluctuations during simulation (Fig. 2B). To avoid inappropriate geometry against steric clashes, minimized energy was ensured as *N*-and *C*-terminal tails fluctuated more than any other part of protein which was seen as a typical observation. Alpha helices and the beta strands (secondary structure elements) fluctuated less when compared to the loop regions (Fig. 2A). The unstructured part of the protein exhibited less rigidity as normalization of the trajectory suggested a 70% interaction during simulation time was maintained. This incorporated a value of 0.7 as few protein residues make multiple contacts of same subtypes with the ligand. The interaction is maintained for all specific interactions which have values above 1.0. The current geometric criteria for H-bond protein–ligand is specific by maintaining a distance of 0.25 nm between the donor and acceptor atoms (D—H···A). The other criterions selected were a donor angle between the donor-hydrogen-acceptor atoms (D—H···A) with a donor angle of ³120° surmounting to the effectiveness of acceptor angle of ³90° between the hydrogen-acceptor-bonded atom atoms (H···A—X). Moreover, the p-Cation; p-p and non-specific interaction hydrophobic contacts encompass aliphatic or aromatic groups on the ligand involving a hydrophobic amino acid. However, this category was extended to include p-cation interactions (Fig. 3A, B) as the docking was based on SrtA-quaternary ammonium substrate complexes and known Sortase-A crystal structure catalyzing amino bonds. Thus, charge-charge interactions inserted inside the binding pocket of Sortase-A was enabled due to the polar capabilities of QAS/k21 molecule.

Figure 3C, D shows Raman spectra and the corresponding section corrected for orientation in a side-by-side image. For better comparability of the two measurements, different colours were chosen for the Raman spectrum of dual specie bacterial biofilms. As expected, Raman spectral image revealed sample features typical of a biofilm signature with characteristically low carbohydrate content (intensities) seen in 1% and 2% QAS specimens (Fig. 3C). The biofilm analysis revealed the strongest Raman spectra between 484 and 490 cm^−1^ belonging to the symmetric vibration of carbohydrates and does not coincide with any of the lines within the frame. The molecules within the aromatic and functional groups have polarized electrons as a result of double bonds and free electrons which resulted in increased Raman shifts inside the specimens (Fig. 3C). The bands refer to the glycosidic link or ring breath of possible polysaccharides which typify the changes seen within the biofilm as a result of disinfectant treatment. There was a striking reduction of the peaks in specimens treated with QAS disinfectant as the concentration reached 2%. Deformation vibrations of single-bond stretch for amino acids at 870 cm^−1^ showed remarkably lesser intensity in 2% CHX specimens as compared to 1% QAS specimens (data not shown). These features are specific for polysaccharides (COC stretching and the anomeric C (1)-H deformations of α (1 → 4) glycosidic links) linked by 1–4 glycosidic bonds (amylose, amylopectin, glycogen). This finger print region attributed to bacterial carbohydrate via CO and CC stretching and bending vibrations showing similar changes as per our previous studies, this time also with 1% QAS molecules.

**Figure 4:**
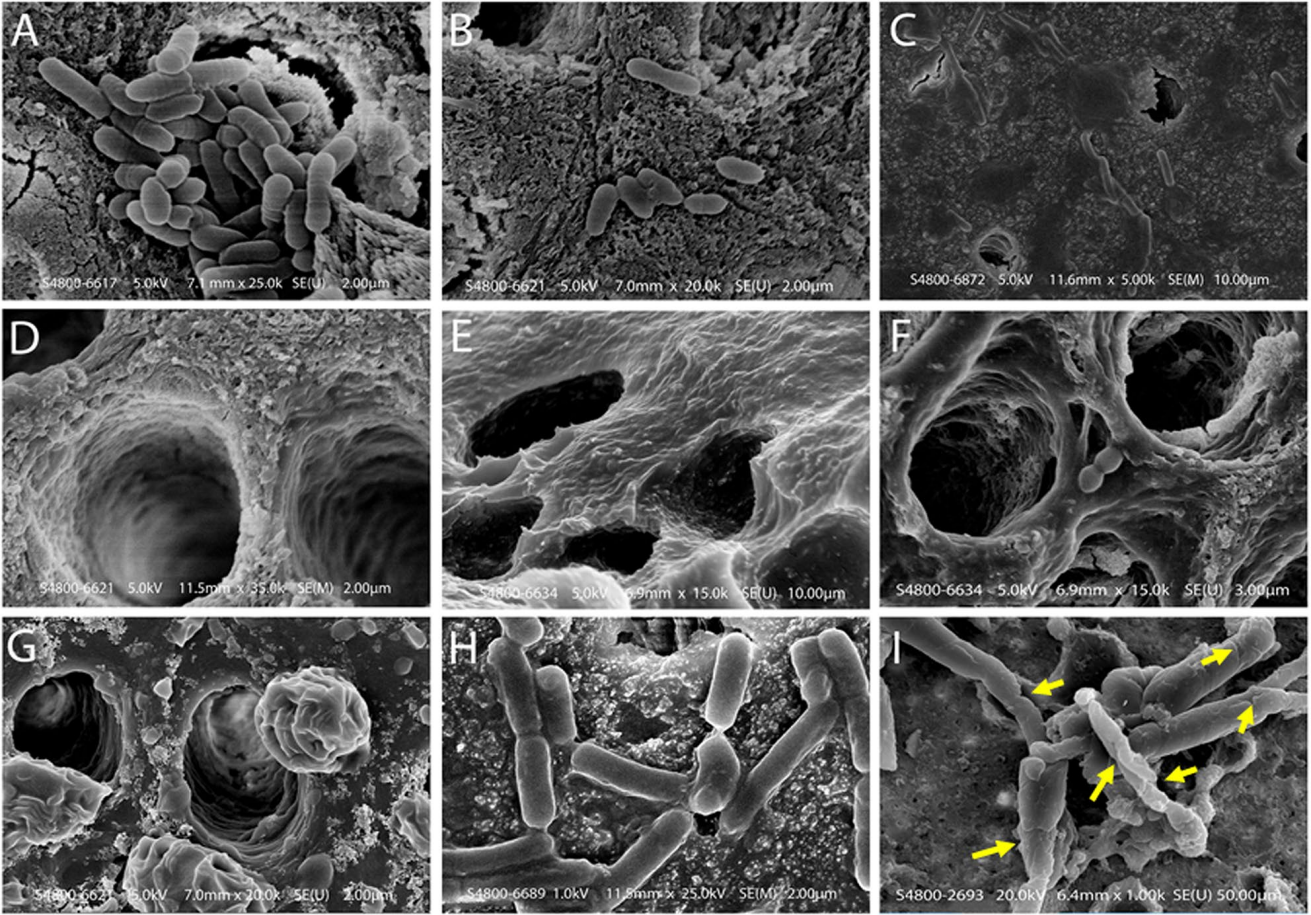
(**A**) Scanning electron microscope of control specimen showing dentinal tubules covered with dual species biofilm. Bacteria and debris are present on the dentine surface without using standard experimental disinfection. Bacteria blocked the opening of the dentinal tubules; groups displayed singular or multiple deposits on the sample with bacterial cells clumping and chaining to form complex biofilms. (**B**) SEM showing incomplete removal of bacteria on the dentine surface after using 2% CHX protocol. These dentinal tubules are located in the middle third of the dentine specimen. There were small colony chain formations seen amongst 2% CHX specimens (Fig. 3B) due to slight restructuring as compared to maximum detachment seen in QAS groups. (**C**, **D**) Bacterial penetration is limited across the lengths of dentinal tubules and dentinal surface demonstrated in 1% and 2% QAS specimens. (**D**) Tubule wall of demineralized dentine treated with 1% QAS shows exposed fibrillar collagen network. (**E**, **F**) Representative SEM images of etched dentine following application of 1% and 2% QAS respectively showing the QAS molecules did not completely infiltrate into the demineralized collagen matrix forming a crust on the surface. A phase separation is seen due to the presence of water (**G**). (**H**) Bacterial biofilms were generally intact within control specimens; (**I**) the bacterial *Lactobacillus* within the biofilm showed rough and wrinkled surfaces observed on the membrane after treatment with 1% QAS. There were large damaged areas including the formation of holes inducing significant damage to the membrane of bacterial cells after use of 1% QAS (**I**).

**Figure 5:**
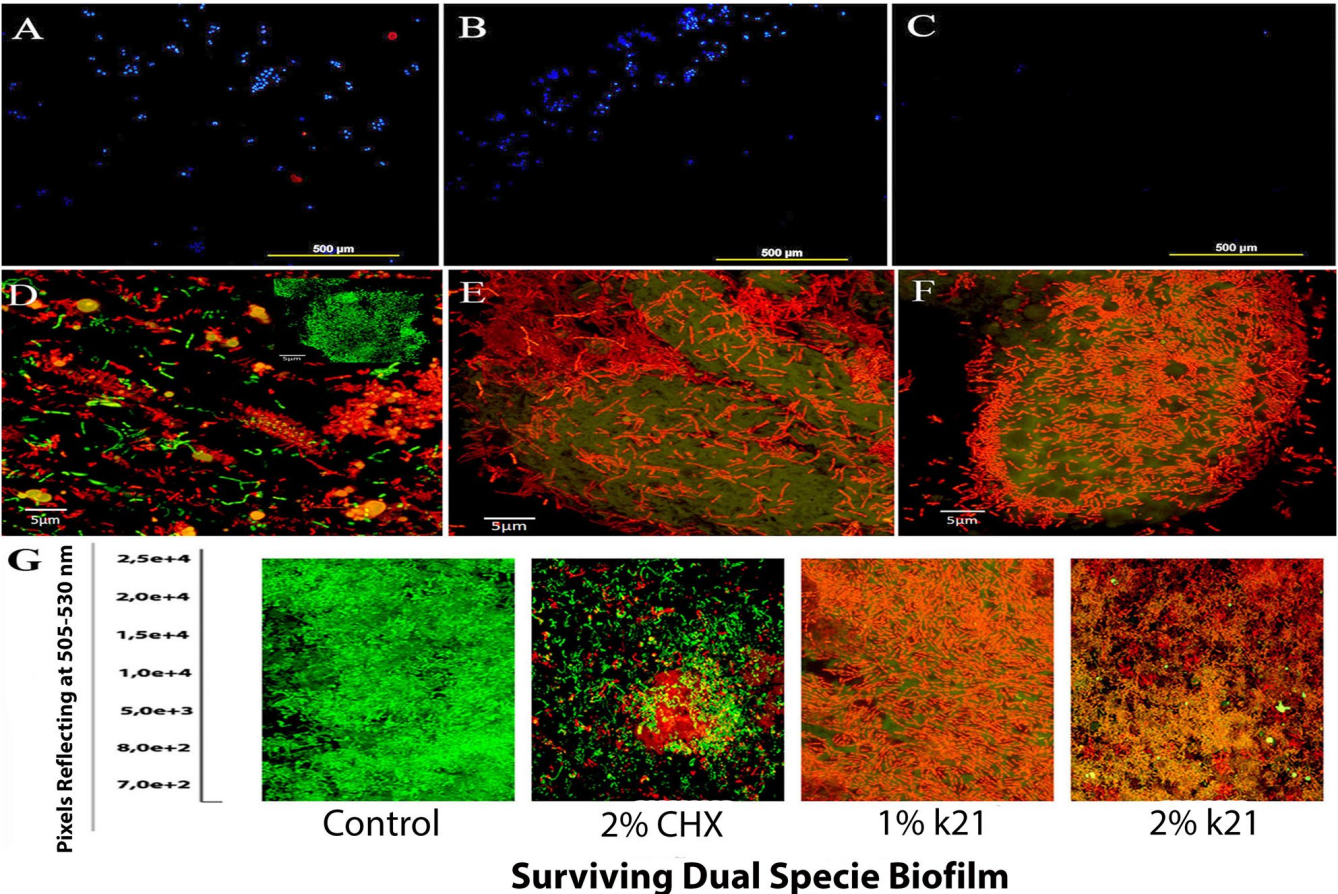
Fluorescent dye 4′,6-diamidino-2-phenylindole (DAPI) assay of (**A**) control, (**B**) 2% CHX and (**C**) 1% QAS specimens; the images shown are consistent with the results of membrane analysis. CLSM images showing (**D**) 2%CHX and dead bacterial sites around the (**E**) 1% and (**F**) 2% QAS molecule attracted due to its surfactant effect. (**G**) CLSM images and viability of dual specie biofilms treated with different antimicrobial agents, after which, biofilms were stained using the BacLight LIVE/DEAD viability stain.

Scanning electron microscopy images of the coronal dentine with and without treatment of disinfectants are shown in Fig. 4. On the other hand, Fig. 4A shows deposition of a thick aggregates of bacteria which has occluded the dentinal tubule within the control specimens. The groups displayed singular or multiple deposits on the sample with bacterial cells clumping and chaining to form complex biofilms. Dramatic changes within the biofilms were found after treatment with 1% (Fig. 4C) and 2% QAS (Fig. 4D) with SEM photographs demonstrating absence of bacterial colonies after treatment. There were small colony chain formations seen amongst 2% CHX specimens (Fig. 4B) due to slight restructuring as compared to maximum detachment seen in QAS groups. There is condensation observed in both 1% (Fig. 4E) and 2% QAS (Fig. 4F) molecules indicating phase separation, as also seen on the surface and inside the dentinal tubules. This phase separation is due to the presence of water (Fig. 4G). As the bacterial biofilms were generally intact within the control specimens (Fig. 4H), the bacterial *L. acidophilus* within the biofilm showed rough and wrinkled surfaces observed on the membrane (Fig. 4I) after 1% QAS treatment. There were large damaged areas including the formation of holes inducing significant damage to the membrane of bacterial cells after use of 1% QAS (Fig. 4I). The images shown are consistent with the results of membrane analysis and the DAPI study. As shown in Fig. 4, great amounts of bacterial cells within the biofilm in the control (Fig. 5A) and 2% CHX (Fig. 5B) groups emitted a blue fluorescence signal. The amount of DAP staining decreased dramatically after treatment with 1% QAS (Fig. 5C). This clearly indicated that less DAPI stained the double stranded DNA within the remaining cells.

Representative CLSM images for dual specie *Streptococcus mutans* and *L. acidophilus* biofilms are shown in Fig. 5D–G. The control specimens showed in the BHI medium (Fig. 5G) showed clusters of green colonies as majority of the dead bacterial cells formed large aggregates and were found in 1% (Fig. 5E–G) and 2% QAS (Fig. 5F, G) specimens. This aggregation was dependent on the concentration and type of disinfectant used (*p* < 0.05). In contrast to the control and 2% CHX (Fig. 5D) specimens, the QAS specimens displayed different characteristics forming thinner biofilms without distinct aggregates suggestive of a dominant role of QAS in removal and non-adherence of bacterial biofilms. The dead bacteria formed dead isolates around the QAS molecule attracted due to its surfactant effect (Fig. 5E, F). The QAS accumulated at the periphery and the biofilm aggregate due to ability of QAS to access the aggregate interior. The cell number was notably increased in control and 2% CHX specimens in contrast to the QAS specimens, a fact that is coincident with the fact that there is incapability to form biofilm (Table 1).

**Table 1:** Statistical significance with post hoc comparisons of different specimens treated with different concentration of QAS molecule and CHX based on Fisher’s protected least significant difference.

	Biofilm Specimens	1	2	3	4	5	6	7	8	9	10
*n* = 3	S Mutans/ Lactobacillus/Control	*p* = 0.065									
		p = 0.094	***p*** = **0.002** **5.18 × 10**^**7**^								
		***p*** = **0.031** **6.24 × 10**^**9**^	***p*** = **0.001**	*p* = 0.154							
*n* = 3	S Mutans/ Lactobacillus/1%QAS	***p*** = **0.05**	*p* = 0.87	***p*** = **0.001**	*p* = 0.673						
		***p*** = **0.003**	***p*** = **0.004**	*p* = 0.431	*p* = 0.299	*p* = 0.555					
		***p*** = **0.023**	***p*** = **0.001**	***p*** = **0.001**	***p*** = **0.034**	***p*** = **0.001**	***p*** = **0.011**				
*n* = 3	S Mutans/ Lactobacillus/2%QAS	***p*** = **0.041**	***p*** = **0.003**	***p*** = **0.001**	***p*** = **0.001**	***p*** = **0.001**	***p*** = **0.001**	***p*** = **0.032**			
		*p* = 0.81	***p*** = **0.42**	***p*** = **0.001**	***p*** = **0.029**	*p* = 0.41	***p*** = **0.001**	***p*** = **0.001**	***p*** = **0.011**		
		*p* = 0.11	*p* = 0.122	***p*** = **0.042**	***p*** = **0.001**	***p*** = **0.001**	***p*** = **0.001**	***p*** = **0.001**	***p*** = **0.001**	***p*** = **0.001**	
*n* = 3	S Mutans/ Lactobacillus/2%CHX	*p* = 0.11	*p* = 0.04	*p* = 0.54	*p* = 0.71	***p*** = **0.05**	*p* = 0.31	***p*** = **0.03**	*p* = 0.54		
		*p* = 0.21	*p* = 0.81	***p*** = **0.01**	***p*** = **0.001**	*p* = 0.42	***p*** = **0.001**	*p* = 0.3			
		*p* = 0.04	*p* = 0.001	***p*** = **0.005**	*p* = 0.6	*p* = 0.62	***p*** = **0.03**				

The bold indicates the statistically significant *p* values.

**Figure 6:**
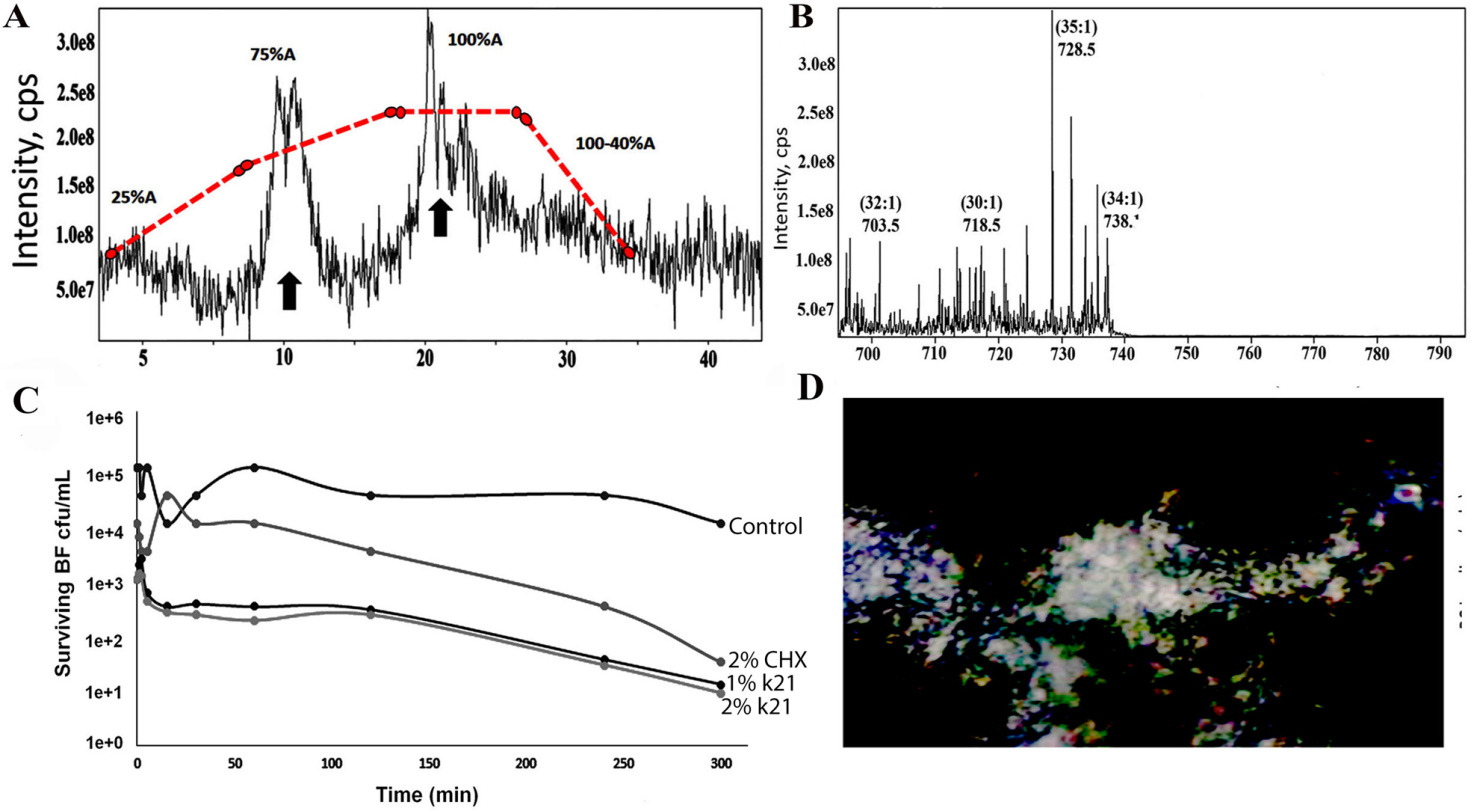
(**A**) Tandem mass spectroscopy analysis of extracted membrane lipids showing effective separation of membrane lipids based on hydrophobicity with the different elution time points. Extracted dual species bacterial organism’s membrane lipids with dotted lines indicated the change in gradient and concentration as QAS percentage was increased to 2% with typical elution time for membrane phospholipids of *Streptococcus mutans* and *Lactobacillus acidophilus* biofilm. (**B**) Tandem mass spectroscopy scan showing different ion modes indicating fragmentation of this species to yield different fatty acyl chains in 1% QAS molecules. (**C**) Time–kill curves of dual specie biofilms treated with different concentrations of antimicrobials. The surviving bacteria were plated at various time points (0–300 min). Both 1% QAS and 2% QAS were able to retard growth for 300 min even after washing during the time-kill assay. After 1 min of QAS treatment, bacterial cells were reduced and showed less survivability. (**D**) Confocal intensity of area of biofilm in 1% QAS specimens under consideration with excitation performed at λ = 514 nM; *QAS* quaternary ammonium silane, *CHX* chlorhexidine, *BF* biofilm, *cps* count per second.

The fatty acid compositions of the dual specie biofilm increased under different DMP concentrations as myristic acid and lauric acid was seen at a rise in both 1% (not shown) and 2% QAS specimens. The contents of fatty acids also increased with a change in the DMP concentration as the SDH activity was also changed significantly (*p* < 0.05). The activity of SDH increased with exposure to 1% and 2% QAS molecules. Both patterns are in accordance to the growth rate (Fig. 6A) showing a significant change in the decrease of fatty acids in 1% and 2% QAS specimens. The dotted lines inside the figure indicated a change in gradient and typical elution time to the membrane fatty acids as the combined ion mass spectra showed a variety of fatty acids. The negative ion mode which was around 34:1 for 1% and 2% QAS specimens, yielded different fatty acid chains, which was another conformational change seen amongst the specimens (Fig. 6B). Selective inhibition of *dual species biofilm* growth was observed after brief exposure to both concentrations of QAS molecule. The cultures were treated for a brief period of time, washed and then transferred to a rich medium. Confocal intensity area of biofilm in 1% QAS specimens was taken under consideration with excitation performed at λ = 514 nm (Fig. 6D). Results indicated (Fig. 6C) that both 1% QAS and 2% QAS were able to retard the growth for 300 min s even after washing. There was a slight increase of bacterial survivability at 125th min, which eventually went down till the 300th min.

## Discussion

A three dimensional silicate network is formed due to hydrolysis of silanol groups in the presence of tetraethoxysilane anchoring unit. There is subsequent condensation of tetra- and triethoxysilane molecules forming Si–O–Si linkages. There is a sol–gel reaction between ethoxysilanes producing ethanol as a condensation product unlike methanol, produced as a result of condensation reaction of methoxysilanes^31^. It has been demonstrated previously that QAS compounds were also effective inside polymeric materials such as quaternary ammonium methacrylate–modified silica nanoparticles, in decreasing bacterial growth^32^. Quaternary ammonium *bis*-phenol A glycerolate dimethacrylate co-polymerized with methacrylate monomers and showed good antimicrobial activity^33,34^. The QAS compound has long lipophilic C18 alkyl chain-like structure inside the sol and network gel consisting of four positively charges. With these four lipophilic arms, QAS attaches to bacteria with negatively-charged cell walls via electrostatic interaction^35^. This penetration of bacterial membranes leads to leakage of cytoplasmic contents and an eventual cell death^36^. The increase in hydrophobic chain length from ~ 12 to 16 units (by changing the chain length of alkyl halide) is imperative to determine a threshold of antibacterial activity^37^. The hydrophobic segment within the chain is compatible with the bilayer of the outer bacterial cell wall, which, on the other hand, has an additional membrane with a phospholipid bilayer structure protecting the inner cellular structure^38^. Increasing the alkyl chain (C18 alkyl chain) of amphiphilic compound increases the antimicrobial activity disrupting membranes as seen in the in-vitro results. The compound has a significant effect against both *Streptococcus mutans* and *Lactobacillus acidophilus* biofilms which are the two most important bacteria associated with dental caries. While studying the dual species model, it enables the analysis of interaction between two species and more complex microbial ecosystems. *Streptococcus mutans* amplifies the cariogenic properties due to sucrose metabolism, adherence and acidogenity via fermentation^39^. In addition to this, *Lactobacilli* are also considered as major contributors to caries progression with activity enhancement in the presence of *Streptococcus mutans* creating a necessary niche for retention of organisms^40^.

The crystal structure of SrtA possess an eight-stranded fold with several loops and two short helices containing β7 and β8 strands forming a hydrophobic floor with amino acids walls^41^. Molecular modelling and simulation was performed to study the relationship between the structure of QAS and its inhibitory activity on SrtA. The ligand torsion plot summarizes the conformational evolution of every rotatable bond (RB) in the ligand throughout the simulation trajectory (0.00 through 100.00 ns). The 3D schematic ligand of color-coded rotatable bonds is shown in the panel (Fig. 2B) with each bond accompanied by bar and dial plots of the same color. The likely targets of quaternary ammonium compounds are cytoplasmic membrane and DNA because they are amphipathic cations. The quaternary ammonium group shows reduced potency of β-hydrastine due to its central ring system^42^. Because of their strong influence on drug specificity, metabolization and adsorption, hydrogen-bonding properties are considered pivotal.

The hydrogen bonds between a ligand and protein can be broken further into four subtypes: side chain donor, backbone donor, side chain acceptor, and backbone acceptor. The docking studies were confirmed using an acceptor angle of ^3^90° between hydrogen-acceptor-bonded atoms (H···A—X). In addition to this, the donor and acceptor atoms (D—H···A) were maintained at a distance of 2.5 nm with a donor angle of ^3^120°. This was all set according to the current geometric criteria for protein–ligand hydrogen bonding. The binding of QAS molecule at both 1% and 2% concentration to SrtA is via electrostatic, hydrogen bond, and ‘van der Waals’ interactions. The strong interaction of QAS with SrT A interferes with the catalytic activity, resulting in reduced anchoring of cell surface proteins (Fig. 2B). On the plot, peaks indicate areas of the protein that fluctuate the most during the simulation (Fig. 1A–C). Sortase A mediated ligation is used for C-terminal modification of protein. This is due to generality, high efficiency, and specificity. On our plot of simulation, peaks indicate areas of the protein that fluctuate the most during the simulation in presence of QAS. Typically, it was observed that the tails (N- and C-terminal) fluctuated more than any other part of the protein. Secondary structure elements like alpha helices and beta strands are usually more rigid than the unstructured part of the protein, and thus fluctuate less than the loop regions. This owes to conformational changes seen as a result of QAS interaction with Srt A membrane protein (Fig. 1).

Biofilms are an interfacial collection of multispecies embedded within a polymeric matrix (extrapolysaccharides) whose growth and maintenance are predominantly sustained by carbohydrates, lipids and proteins found inside the matrix. This serves as a communication medium (quorum sensing) and acts as a supply of energy^43^. In light of this, there is a need to develop a deeper understanding of bacterial diversity and the inhibitory effects of chemical agents in order to identify and develop appropriate biocides and workable strategies in dentistry. Now, CLSM at times may lack the desired penetration depth and specificity required while doing analysis as the staining method maybe difficult to engage for diverse polymeric materials^44^. By contrast, spectroscopic analysis using the Raman method provides a holistic, rapid, noninvasive qualitative and quantitative analysis with characteristic vibrational modes arising from different parts of the EPS matrix, especially the carbohydrates^45^. In our current study, the signature vibrational mode seen in the region of 480–490 cm^−1^ can be assigned to carbohydrates and polysaccharides^20, 46^. It is observable that different concentrations of QAS showed deviations and differences within the Raman spectra with the least intensity seen amongst 2% QAS specimens. The peaks appeared dull suggestive of bacterial colonies getting affected at both concentrations of QAS disinfectant; further confirming the comprehensive effect even at a lower concentration of 1%. Similarly, the deformation single-bond stretch vibrations for amino acids also displayed lesser signals in both the specimens. Therefore, it is possible that Raman spectroscopy detected the difference in the quality of biofilms or even the difference in the cell walls of bacteria which also includes polysaccharides^47^. A sharp band around 497 cm^−1^ also represents the capsular polysaccharides found in gram positive bacteria^48^.

The presence of biofilm and morphological changes of bacterial cells were observed using SEM. The effect of QAS both at 1% and 2% concentrations lead to an increase in surface hydrophobicity. This is due to a change in the energy substrate of the demineralized dentine surface (Fig. 3D, E). This in turn is a direct measure of increased wettability^49^. Organofunctional silanes react with water molecules of the dentine surface due to its scavenging effect converting the hydrolysed alkoxy groups of the silanes to alcohol molecules^50^. Results from the SEM study have shown that QAS has a significant effect on the biofilms at both concentrations. Wrinkled areas were observed on the bacterial cells proving a significant effect on the bacterial biofilms. Most of the attention in the past regarding caries was given to the use of CHX with variable outcomes while reporting differences^51^. There are many reactive silanol groups generated as a result of hydrolysis resulting in a covalent attachment of QAS molecules to the substrate via Si–O linkages to exert non-migrating microbial functions^27^. This enables a protective layer formed on top of the substrate as seen in the Fig. 4E. These are stable non-polar and hydrophobic structures modifying the surface with QAS condensation on the surface and inside the tubules be seen as spherical bodies (Fig. 4G). In the present study, the DAPI method was used to further assess the integrity of bacterial cells or DNA damage. This was further validated by observing bacterial cells after DAPI staining as shown in the figure. Great amount of bacterial cells showed blue nuclei in the control group as compared to the 1% and 2% QAS group DAPI staining. This suggested that less DAPI stained double-stranded DNA remained in the cells giving a clear indication of DNA and other intracellular contents leaking from the damaged cell membrane in specimens treated with QAS. This membrane damage induced by QAS at both concentrations is considered pivotal resulting in massive discrepancy and bursting of the bacterial cell due to osmotic pressure^52^. In addition, QAS has strong anti-protease activities against MMPs responsible for degradation of demineralized collagen fibrils^15^.

The use of tetrafunctional organosilane acts as trialkoxysilane anchoring units which form three dimensional networks as condensation occurs^53^ on the dentine surface. This provides a longer lasting antimicrobial activity primarily due to quaternary ammonium molecules minimal leaching from the surface and covalent attachment in the presence of the silanol groups. There is a bond formed between the nucleophile and the silicon atom during hydrolysis causing self-condensation as mentioned previously. The free silanol groups form hydrogen bonding with the hydroxyl groups, with further formation of –Si–O–(substrate)-linkage between the HO-(substrate) and the silanol^54^. This also leads to surfactant activity reducing the microbial adherence and slowing down biofilm formation. The current study demonstrates that the viability of biofilms is affected by the QAS used, which stays independent of the loss of surface layer^55^. The study mentioned, regarding QAS at different concentrations, has justified its significant potential for future commercialization for dental applications. Results of CLSM obtained clearly suggest a debilitating effect of QAS against dual specie bacterial biofilm. The log difference that was calculated for bacterial counts openly suggested a statistical difference amongst different biofilm groups according to the Fisher’s protected least significant difference.

The activity of SDH was changed as a result of DMP which may result in alteration of the energy metabolism in the membrane of dual specie biofilms. The outer cell membrane has an all important role of maintaining cell culture and ensuring transport of materials in and outside^56,57^. The expression levels of SDH and fatty acids were increased which could have been caused by the activation of killing mechanisms of QAS molecules. The membrane of dual species was damaged by QAS and was concentration dependent. It certainly explains our observations and results that QAS exerted on the biofilm. Hence, it was conjectured and analyzed that cytoplasmic membranes of bacteria are the critical targets. Having established the fact that the QAS molecule had improved antimicrobial properties in mononuclear conditions, we sought to determine if QAS was able to exhibit the same effectiveness in a dual specie mode and at a much lower concentration. After 1–2 min of QAS treatment, the cells were selectively killed also in mixed cultures and is therefore capable of eliminating targeted species within the mixed culture system.

## Conclusion

The QAS/k21disinfectant is effective and ensures a significant decrease in the microbial growth. The significant destruction of bacterial membranes and understanding of the conformational change of QAS towards Sortase A attachment during strongly correlates to the Raman vibrational and confocal changes. The results of 1% QAS/k21 antimicrobial action suggest a greater potential for development of safer disinfectants and a biofilm inhibitor as compared to its previous efficacy of 2% QAS.

## Materials and methods

The current study design was approved by the Joint Committee on Research and Ethics (1-01/2019/425/2018) at International Medical University. All research was performed in accordance with relevant guidelines/regulations and informed consent was taken from all patients who had donated the extracted teeth for the experiments. Experimental versions of the QAS used in the present study were synthesized by sol–gel reaction and generously provided by KHG FiteBac*®* Technology. The QAS is prepared from cationic 3-(trimethoxysilyl)-propyldimethyloctadecyl ammonium chloride (SiQAC; C_26_H_58_ClNO_3_Si; CAS Number 27668-52-6)^58,59^ and tetraethoxysilane (TEOS) via sol–gel reaction to produce a host of molecules with different molecular weights that are collectively code-named k21/QAS (CAS Registry Number 1566577-36-3; IUPAC name: 1-octadecanaminium, *N,N*′-[[3,3-bis[[[3-(dimethyl octadecylammonio)propyl]dihydroxysilyl]oxy]-1,1,5,5-tetrahydroxy-1,5-trisiloxanediyl]di-3,1-propanediyl] bis-[*N,N*-dimethyl] chloride (1:4))^60,61^. The fully-hydrolyzed, partially-condensed series of molecules are dissolved in ethanol or acetone to produce a cavity disinfectant for intraoral use. Attenuated total Reflection-Fourier transform infrared spectroscopy (ATR-FTIR; Thermo Fisher Scientific, USA) was performed with multiple reflections to monitor the hydrolysis and condensation of ethoxysilanes. Spectra were obtained 650–4,000 cm^−1^ range at a resolution of 4 cm^−1^ and an entrance angle of 40° (Supplementary Fig. 1). Sound human molars (*n* = *90*) (from patients aged between 23 and 34 years old) were stored after extraction in a solution of deionized water and sodium azide (0.2%) at 4 °C to inhibit microbial growth and were used within 1 month from the start of the experiments. QAS and other experimental groups were randomly assigned into following four; Group I = Control; Group II = 2% chlorhexidine (CHX); Group III = 1% QAS; and Group IV = 2% QAS. To standardise the application each time in all groups, the disinfectants were applied using a sterile saturated micro brush (Dentsply DeTrey, Konstanz, Germany) and rubbed for 20 s. The specimens were returned to the well plates for experimentation.

### Sortase A activity inhibition assay

Cleavage of a synthetic fluorescent peptide substrate, such as 4-(4-dimethylamino phenylazo) benzoic acid (Dabcyl)-QALPETGEE-5-[(2-aminoethyl) amino] naphthalene-1-sulfonic acid (Edans) (Dabcyl-QALPETGEE-Edans) (Jiershenghua, Shanghai, China) was used to analyze the inhibition of Sortase A by QAS through quantifying increased fluorescent intensity. 300 μL of reaction buffer (50 mM Tris–HCl, 5 mM CaCl2, 150 mM NaCl, pH 7.5) was used with purified sortase A (5 μM) for reactions with fluorescent peptide substrate (10 μM), NH_2_OH (0.2 M), and varying concentrations of QAS. Sterilized distilled water was used to dilute different concentrations of QAS in 1% dimethyl sulfoxide (DMSO). Appropriate number of blanks were used. For positive control, ρ-hydroxymercuribenzoic acid (pHMB; Sigma Aldrich) was used. The reactions for fluorometric analysis (SpectraMax Gemini XS, USA) were carried out for 1hour at 37 °C with an excitation wavelength of 450 nm recordings. All experiments were performed in triplicates.

### Molecular docking simulations (antibacterial agent on Sortase A)

The small-molecule drug discovery suite (Schrödinger, USA; Version 2018-2) was used to obtain insights on binding of the quaternary ammonium silane/QAS to the active sites on Sortase A at the molecular level. The crystal structure of *Streptococcus mutans* Sortase A (PDB ID: 4TQX) was downloaded from Research Collaboratory for Structural Bioinformatics Protein Data Bank (PDB; https://www.pdb.org). A computer model of the inner bacterial membrane was built from the phospholipids palmitoyl-oleoyl-phosphatidylethanolamine (POPE) and palmitoyl-oleoyl-phosphatidylglycerol (POPG) in a 3:1 ratio (10.16 × 7.21 × 14.44 nm^3^). All atoms in the QAS were restrained in space with the QAS plane perpendicular to the membrane surface and with the most inferior atoms 0.9 nm above the membrane surface. The ‘protein preparation’ module of Schrödinger with default settings was used to prepare the protein for docking. Water molecules with less than three hydrogen bonds were removed at the corresponding pH of 7.0 as the protein was prepared. Missing loops and side chain atoms were added in the protein structure with energy minimized using OPLS_2005 force field. The active sites in Sortase A were determined using ‘SiteMap’ module of Schrödinger with default settings as bacterial proteins. The chemical structure of the quaternary ammonium silane was sketched using Maestro 11.8 and 3D structures prepared using the Ligprep module and OPLS_2005 force field. This optimized the low-energy conformers of all vanilloids which were docked into the binding site using the extra precision (XP) mode which incorporated water desolation energy and protein–ligand structural motifs, and enabled the binding free energy scoring function.

### Micro-Raman spectroscopy

Designated dentine disks were removed from the culture plates and dried for 15 min at 35 °C. Each dentine disk was transferred to a low fluorescent quartz microscopic slide. The latter was placed on an x–y–z-axis-positioning stage. Areas of 20 µm were randomly chosen from each air-dried biofilm for micro-Raman spectroscopy. A Leica microscope equipped with lenses (JY LabRam HR 800; Horiba Jobin Yvon, France) and curve-fitting software (Labspec 5, Horiba) was employed for analysis. The following parameters were employed for spectrum acquisition using a 100 × objective: 514.5 nm green laser excitation, 785 nm with argon ion (spectral resolution 1.6 cm^−1^), < 500 μW. Ten frames of 20 s each were recorded for each biofilm specimen. Data were subjected to background and noise removal with dark count correction. All spectra were normalized to the following peaks and spectral distance calculated using the OriginPro 8.5.1 software (Origin Lab Corp., USA): 1,454 cm^−1^ (CH_2_ deformations)^62^, 1655 cm^−1^, 1,245 cm^−1^ and 1,070 cm^−1^ for biofilm changes. The Rayleigh scattering photons were blocked with a notch filter that has a spectral range of -120 to 130 rel.cm^−1^, with an ellipsoid measurement of approximately 1 µm. The spatial resolution in the horizontal plane was 350 nm and 2.0 µm. Raman peaks were also centered at 1,454 cm^−1^ (pyrrolidine rings of proline and hydroxyproline inside collagen), 1655 cm^−1^ (amide I [C = O])^63^, 960 cm^−1^ (hydroxyapatite PO_4_), 434 cm^−1^ (stretching vibration of ν2PO4)^64^ and 484 cm^−1^ (polysaccharides or carbohydrates)^65^. The specimens were inspected visually using light micrography before and after Raman measurements to ensure that the biofilms were not submerged or damaged during analysis, which could have generated errors.

### Bacterial strains, culture media and growth conditions

Dual-species biofilms were cultured on acid-etched dentine disks according to the previously published protocol^20^. Intact, non-carious human third molars (from donors aged 21–34 years) were collected after obtaining patient’s informed consent. The occlusal enamel of each tooth was cut perpendicular to its longitudinal axis with a low-speed diamond saw (Isomet, Buehler Ltd., Lake Bluff, IL, USA) with water cooling, to expose the mid-coronal dentine at 1 mm below the dentinoenamel junction. The dentine disks were wet-polished with 1,200-grit silicon carbide paper, acid-etched with 1% citric acid for 5 min to remove the smear layer and smear plugs. Later teeth samples were rinsed with deionized water and autoclaved at 121 °C for 15 min.

*Streptococcus mutans* (ATCC 35,668) and *Lactobacillus acidophilus* (ATCC 4,356) (American Type Culture Collection, Manassas, VA, USA) were used at a ratio of 60/40 for culturing dual-species biofilms. The bacteria were first cultured separately in Brain Heart Infusion broth (MilliporeSigma, Burlington, MA, USA) that was supplemented with 8% sucrose and 2% xylitol (pH 7.4). Culture was performed anaerobically (10% hydrogen, 5% carbon dioxide and 85% nitrogen) at 37 °C for 48 h. The bacterial suspensions were mixed to yield a bacterial population of 10^7^ colony forming units (CFU)/mL. A 300 μL aliquot of the mixed bacterial suspension was added to each dentine disk. The latter was positioned in a sterile 24-well tissue culture plates and incubated for 120 min at 37 °C in an orbital shaker incubator at 75 rpm to facilitate bacterial adhesion on the dentine disk. Biofilms were allowed to grow for 3 days under anaerobic conditions, with the medium replaced daily. After 3 days, the surface of each dentine disk was treated with the designated disinfectant (experimental QAS or control) using a microbrush and left for 20 s. The dentine disks were returned to the culture medium and cultured for four more days.

### Scanning electron microscopy and DAPI analysis

Exposed surfaces of dentine disks that contained with dual-species biofilms were acid-etched with 32% phosphoric acid (Uni-Etch, Bisco Inc., Schaumburg, IL, USA) for 15 s, rinsed with deionized water and kept visibly moist. The teeth were exposed to 2% CHX (Aplicare, Meriden, CT, USA), 1% QAS or 2% QAS using a sterilized microbrush, left undisturbed for 20 s and gently air-dried (*n* = *5*). The effect of QAS application on etched dentine was examined using scanning electron microscopy (SEM) to evaluate the interaction of the antibacterial agent with the dentine surface.

Additional dual-species biofilms were centrifuged at 4,000 G for 15 min during the logarithmic growth phase. The collected pellets were washed with phosphate buffered saline (PBS; pH 7.0) five times and fixed with 2.5% glutarldehyde for 4 h at 4 °C. The pellets were then dried with ascending grades of ethanol (25%, 35%, 45%, 75%, 100%), critical point dried and sputter-coated with gold. Morphological changes within individual bacterium and colonies were analyzed using SEM (Hitachi S-3400 N, Hitachi High Technologies America, Greenville, SC, USA) at 10 kV. Cell features were compared with cells from the untreated control.

Additional biofilm specimens (n = 3) were centrifuged for 4,000 g for 15 min and rinsed with PBS. The specimens were incubated with 25 µg mL^−1^ of Vectashield® DNA-binding 4′,6-diamidino-2-phenylindole (DAPI) solution (H-1200; Vector Laboratories, Burlingame, CA, USA) for 5 min in the dark at ambient temperature. Analysis of the blue fluorescence within the biofilms was performed using CLSM (Fluoview FV 1,000) at 358_(excitation)_⁄461_(emission)_ nm.

### Confocal microscopy

After the bacterial adhesion phase, the dual-species biofilms were examined with a confocal laser scanning microscope (CLSM; Fluoview FV 1,000, Olympus, Tokyo, Japan). The biofilms were stained with a Live/Dead® BacLight™ Bacteria Viability Kit (Thermo Fisher Scientific, USA) in the dark for 15 min and examined using excitation/emission wavelengths (nm) of 485/498 for SYTO® 9 (green fluorescence for live bacteria) and 535⁄617 for propidium iodide (red fluorescence for dead bacteria). Ten images, each with an area of 212.34 µm × 212.34 µm, were acquired from each double combination of SYTO® 9 and propidium iodide fluorophore-stained biofilm using a 0.5 μm step size. The acquired data were analyzed with the BioimageL software (v.2.0. Department of Oral Biology, Malmö University, Sweden) based on color segmentation algorithms to generate the respective percentages of live and dead bacteria within the biofilm.

### Fatty acid extraction and succinic dehydrogenase assay

Fatty acids in the bacterial membranes of the dual-species biofilms were extracted at the logarithmic phase and exposed to various concentrations of the antibacterial agent used in the present study. The bacterial cells were collected by centrifugation at 4,000 rpm and washed three times with PBS. NaOH-methanol, at a concentration of 15%, was added to the bacterial cells and heated for 5 min in a boiling water bath. The mixtures were saponified with 25% HCl-methanol. Hexane/methyl *tert*-butyl ether was used to extract the mixtures, which were concentrated using nitrogen gas. The fatty acid esters obtained were quantified using a MAT 90 double-focusing mass spectrometer (Finnigan MAT, San Jose, CA, USA). The lipid extracts were centrifuged (3,000 rpm) for 10 min and a 5 µL aliquot was transferred to the electrospray capillary. The temperature was raised to 250 °C at a rate of 10 °C/min. Parameters used for the mass spectrometer were: 50–550 mz^−1^ at 70 eV; ± 700 V was applied to the capillary for detection of positive and negative ions. Twenty repetitive scans were performed for a duration of 5 s in presence of argon collision gas at 3 mTorr.

For succinate dehydrogenase analysis, the dual-species biofilms were harvested at a 5.5 h logarithmic phase using centrifugation (10,000 rpm). After washing the cell pellets five times with PBS, the cells were re-suspended in distilled water and disrupted using ultrasonic cell disruption (JYP-1200L, Zhixin Instrument, Shanghai, China) for 15 min. Centrifugation was performed at 6,000 rpm for 15 min at 4 °C to remove cell debris and cell walls. A Succinate Dehydrogenase Activity Colorimetric Assay Kit (ab228560; Abacm, Cambridge, MA, USA) was used to examine the supernatant for the activity of this enzyme complex.

### Time-kill assay

A time kill assay was performed by growing bacteria in the log phase and treating them with 1 and 2% QAS disinfectant concentrations. Dual-species biofilms were diluted to 10^5^ CFU/mL inside the growth medium. Different disinfectants (2% CHX, 1% k21, 2% k21) were added to the bacterial suspensions in each sample and adjusted to 32, 64, and 128 µg/mL, with no disinfectant serving as control. Ten microliter of the suspension was collected at 0, 1, 2, 5, 15, 30, 60, 120, and 240 min, with further dilution (1:50) and growth-halted by keeping cell suspension with ice. The collected suspensions were then spread on growth medium agar plates, keeping 80 CFU/ml as the detection limit for the assay analysis. Colonies were counted after culturing for 24 h at 37 °C under anaerobic conditions. The experiment was performed in triplicates with three independent experiments and a graph plotted against time.

### Statistical analysis

Data were analyzed one-way analysis of variance (ANOVA) and the post hoc Fisher’s least-significant difference analysis for multiple comparisons at α = 0.05.

## Supplementary information

Supplementary Video.

Supplementary Information.
