# Supplementary material for: A quaternary ammonium silane antimicrobial triggers bacterial membrane and biofilm destruction
Source: Sci Rep. 2020 Jul 3;10:10970. doi: 10.1038/s41598-020-67616-z (PMC7335202; doi:10.1038/s41598-020-67616-z)
Supplement: Supplementary file 2 — Supplementary Information. [file 41598_2020_67616_MOESM2_ESM.docx]

**A Quaternary Ammonium Silane Antimicrobial Triggers Bacterial Membrane and Biofilm Destruction**

**Umer Daood^1*^, Jukka P Matinlinna^2^, Malikarjuna Rao Pichika^3^, Kit-Kay Mak^3^, Venkateshbabu Nagendrababu^1^, Amr S Fawzy^4^**

^1^ Clinical Dentistry Division, School of Dentistry, International Medical University Kuala Lumpur, 126, Jalan Jalil Perkasa 19, Bukit Jalil, 57000 Bukit Jalil, Wilayah Persekutuan Kuala Lumpur, Malaysia

^2^ Dental Materials Science, Applied Oral Sciences & Community Dental Care, Faculty of Dentistry, The University of Hong Kong, 34 Hospital Road, Sai Ying Pun, Hong Kong SAR, PR China

^3^ Department of Pharmaceutical Chemistry, School of Pharmacy, International Medical University Kuala Lumpur, 126, Jalan Jalil Perkasa 19, Bukit Jalil, 57000 Bukit Jalil, Wilayah Persekutuan Kuala Lumpur, Malaysia

^4^ UWA Dental School, University of Western Australia, Nedlands, WA 6009, Australia

***Corresponding Author:** Clinical Dentistry, Restorative Division, Faculty of Dentistry, International Medical University Kuala Lumpur, 126, Jalan Jalil Perkasa 19, Bukit Jalil, 57000 Bukit Jalil, Wilayah Persekutuan Kuala Lumpur, Malaysia

Email: umerdaood@imu.edu.my; Tel: +601151664374


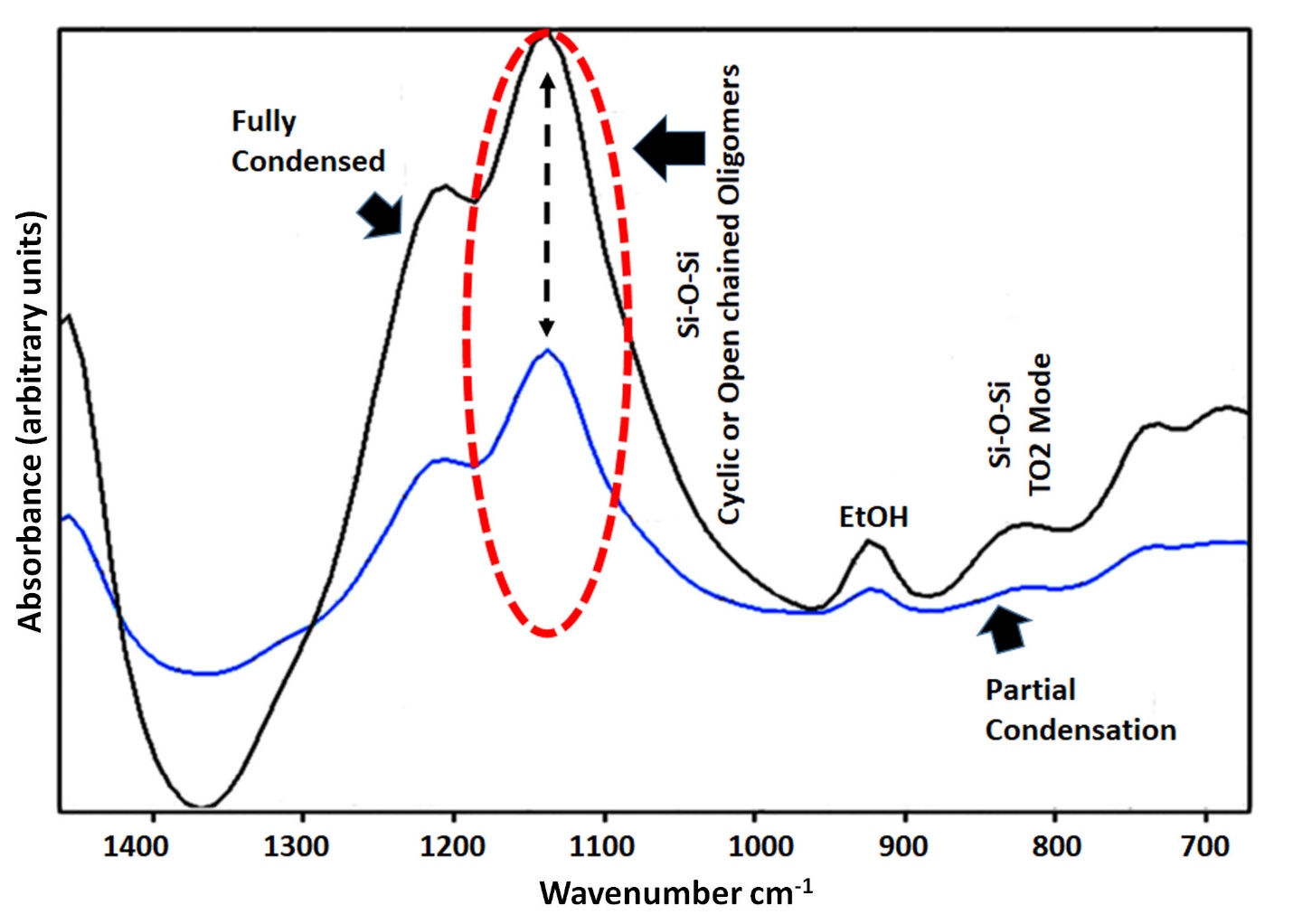


Supplementary Figure 1: Infrared spectra of QAS/k21 showing the hydrolysis reaction between TEOS and Et-SiQAC; the comparison between the hydrolysis reaction products between the partially and fully condensed k21 QAS.
